# A global health crisis in the making: the US withdrawal from the World Health Organization and its impact on global health equity

**DOI:** 10.7189/jogh.15.03043

**Published:** 2025-10-03

**Authors:** Esteban Ortiz-Prado, Josh West, Jorge Vasconez-Gonzalez, Juan S Izquierdo-Condoy

**Affiliations:** 1 One Health Research Group, Faculty of Health Sciences, Universidad de Las Américas, Quito, Ecuador; 2 Brigham Young University, Provo, Utah, USA

## Abstract

The USA initiated their year-long withdrawal from the World Health Organization (WHO) in January 2025, citing financial and governance concerns. This could have a significant impact on global health, as the country is the WHO’s largest financial contributor, providing USD 1.284 billion in 2022–23 and funding crucial initiatives like pandemic preparedness and health equity programmes. Its exit creates uncertainty for the organisation, potentially delaying responses to global health crises and exacerbating inequities, particularly in low- and middle-income countries reliant on US-supported health programmes. Vulnerable populations in Africa, Southeast Asia, and Latin America, for example, face challenges in addressing pandemics and diseases like HIV/AIDS, malaria, and tuberculosis. The USA’s move underscores the fragility of global health governance, raising concerns over the WHO’s ability to lead on critical issues without its support. To mitigate these effects, the international community must adopt innovative, collaborative funding models to maintain the organisation’s mission, emphasising global solidarity and political neutrality to ensure stability, health equity, and robust responses to future health threats.

In January 2025, the US President Donald Trump signed an executive order withdrawing the country from the World Health Organization (WHO), citing issues such as mismanagement during the COVID-19 pandemic to the country bearing a disproportionate financial burden compared to other Member States [1]. While the decision has sparked debates on the organisation’s future, it also raises a fundamental question: what are the broader implications for global health, particularly in the context of health equity and the collective fight against emerging global health threats?

The WHO has been instrumental in coordinating responses to global health crises such as pandemics, natural disasters, and emerging infectious diseases [2]. Its leadership has been essential in the development of frameworks for health emergencies and achieving the Sustainable Development Goals, particularly in reducing global health inequities [3,4]. Yet, the USA’s decision to withdraw calls into question the future of such collaborative efforts. 

The USA has historically played a predominant role in global health financing, serving as the largest contributor of development assistance for health for several decades. Its influence on the WHO is particularly notable, as its financing accounted for 19.77% and 13.96% of WHO expenditures in 2022 and 2023, respectively [5]. During this period, the USA emerged as the WHO’s top donor, contributing an impressive USD 1.284 billion in the 2022–23 biennium. This financial support, along with contributions from other donors, helps fund global health initiatives like pandemic preparedness, emergency responses, and health security measures. Its withdrawal could thus leave a significant gap in the funding needed to sustain these efforts. Various programmes are being affected, such as the Global Fund to Fight AIDS, Tuberculosis, and Malaria, or Gavi, the Vaccine Alliance, and there will also be an impact on disease surveillance and pandemic response [6,7]. The WHO has long depended on the USA for financial backing, and this decision may force it to seek alternative funding sources that may not provide the same level of support, potentially delaying critical responses to global health threats [8]. Withholding US participation, which accounts for a significant portion of the WHO’s funding, could lead to instability within the organisation, potentially impacting its ability to respond to future health crises equitably.

## THE US WITHDRAWAL: A STEP BACK FOR GLOBAL HEALTH?

Furthermore, President Trump’s reasoning that the WHO has been too influenced by political powers such as China highlights concerns about transparency and accountability in global health governance [9]. It might also lead to global discussions about political neutrality, triggering a response of decoupling health issues and politics. This debate over political neutrality inevitably raises a broader concern: without USA’s participation, will the WHO retain the credibility and capacity needed to lead on critical issues such as vaccine distribution, pandemic preparedness, and the fight against antimicrobial resistance?

While the full extent of investment by the USA in the WHO to combat antimicrobial resistance is not known, it is known that the US Centers for Disease Control and Prevention (CDC) alone has allocated nearly USD 2.2 billion in annual and supplemental funding to antimicrobial resistance partners since 2016 [10]. Among its investments are the funding of over 800 projects, a USD 650 million investment aimed at addressing knowledge gaps with scalable solutions, the distribution of USD 57 million through the Broad Agency Announcement, and the fulfilment of more than 5600 isolate panel orders through the Antimicrobial Resistance Isolate Bank [11].

Without US leadership and expertise, the WHO’s ability to carry out its crucial work, such as eradicating polio, combatting antimicrobial resistance, and preparing for future pandemics, will likely be curtailed significantly. The USA has been an active participant in the organisation’s work on health surveillance, biolab safety, and global health security [12]. With its withdrawal, the WHO will almost certainly find it more challenging to engage in the essential collaborative efforts required to ensure health security worldwide.

## IMPACT ON HEALTH EQUITY

One of the most pressing concerns with the USA’s withdrawal is its potential to exacerbate existing health inequities. The WHO has been at the forefront of efforts to improve health equity, working towards a world where healthcare is accessible to all [4]. The USA’s exit could hinder such efforts globally, particularly for vulnerable populations in low- and middle-income countries. The WHO’s initiatives to reduce disparities in health outcomes, such as maternal and child health, HIV/AIDS, and tuberculosis, might face increased challenges without the financial and political support of the USA [8].

The withdrawal also places further strain on the world’s poorest regions, especially countries in the Global South. Many of these nations already struggle with inadequate healthcare systems, insufficient resources, and limited access to essential services [13]. The USA has historically played a key role in supporting these countries through financial contributions, technical assistance, and emergency response programmes [14]. In its absence, they could find it even more difficult to manage emerging health threats like pandemics, which disproportionately impact vulnerable populations. In short, the USA’s exit may leave many countries, especially in Africa and parts of Asia and Latin America, without the support they need to tackle these challenges effectively.

## A GROWING DIVIDE: THE STRAIN ON LOW- AND MIDDLE-INCOME COUNTRIES

Developing countries, particularly those that rely heavily on international aid, will bear the brunt of the USA’s withdrawal. The lack of its support in global health frameworks, including those coordinated by the WHO, will make it significantly harder for these nations to prepare for and respond to future pandemics [15]. As seen in past global health emergencies, a lack of a coordinated global response tends to worsen the crisis in less-developed regions.

Many low- and middle-income countries are already struggling to build and maintain strong health systems, and without the support from major players like the USA and the WHO, they will be left vulnerable to outbreaks and health emergencies [16]. Countries in Africa, Southeast Asia, and Latin America that were once recipients of US assistance in fighting epidemics like Ebola and Zika will find it harder to implement effective public health strategies without the resources they once received [17]. The fight against diseases that disproportionately affect these regions will become more difficult as US-funded programmes, such as those spearheaded by the President’s Emergency Plan for AIDS Relief (PEPFAR) and the United States Agency for International Development (USAID), are curtailed [18].

## THE CASE OF USAID AND PEPFAR

The USAID managed an annual budget of USD 42.8 billion in 2023, equivalent to 42% of global humanitarian aid [19]. Its dismantling has affected a wide range of humanitarian programmes, including food security, nutrition, and emergency relief initiatives; in 2024 alone, for example, it allocated around USD 24 million to the United Nations World Food Programme [20]. Due to its dismantling, it is estimated that approximately 25% of all international cooperation programmes will be canceled. Such is the case of Save the Children, which has been forced to halt much of its operations; according to its data, around 200 000 people in Congo have lost access to healthcare, 50 000 refugee children in Tanzania can no longer attend school, and 85 000 children in Somalia will no longer receive treatment for malnutrition [21].

Sexual and reproductive health programmes, as well as gender equality initiatives, have also been affected. Around two million women and girls have been unable to access contraceptive treatments, and estimates suggest that in just 90 days, there could be up to four million unintended pregnancies and at least 8000 women dying in childbirth [22]. Programmes that carried out campaigns to combat Ebola and polio have also been affected, as have projects focussed on access to clean water, food, and education [21].

The PEPFAR, meanwhile, receives 20% of its budget through USAID [23]. The freezing of funds affects programmes that distribute HIV medications in dozens of countries; there has, for example, been a significant decline in community-based HIV prevention initiatives, reduced adherence to treatment, and cutbacks in counselling, support, and social follow-up activities [21,24]. The WHO has indicated that the disruption of these programmes exposes people living with HIV to an immediate increased risk of infection and death, while also undermining efforts to prevent its transmission [25]. Several countries are already experiencing the consequences of this decision: PEPFAR-funded projects supporting HIV comprehensive care clinics in El Salvador have been suspended; the distribution of pre-exposure prophylaxis in Guatemala, a preventive medication for HIV, has been interrupted; while in Haiti and Jamaica, where PEPFAR funds cover 60% and 50% of services, programmes have also been severely impacted [24].

## POTENTIAL ALTERNATIVES: CAN WE AFFORD TO GO WITHOUT THE US?

While the USA’s withdrawal may not immediately incapacitate the WHO, the organisation may have to look for alternative sources of funding and support. In this sense, it will have to explore innovative models of collaboration that do not rely on any single nation, especially one as influential as the USA. The global community, including philanthropic organisations, regional health networks, and emerging economies, must step up their roles to fill the void left by the USA.

Efforts like the WHO’s Investment Round, where Germany and other countries pledged significant funding in 2024 to support the organisation's mission, will need to become more widespread [26]. Tania Cernuschi, an employee of the WHO, has launched the campaign '1 Dollar, 1 World', which has raised approximately USD 70 076.95 [27]. It is necessary for other powers to fill the void left by the US government; the European Union and China are the ones with the greatest potential to do so [28]. The BRICS nations (Brazil, the Russian Federation, India, China, and South Africa), whose economies have achieved significant improvements in health outcomes, progress toward the Millennium Development Goals, and notable advances toward universal health coverage and the strengthening of their health systems, could play an important role as contributors to the WHO [29].This collaborative and more diversified funding approach is essential to maintain the organisation’s political neutrality, financial independence, and capacity to address global health threats equitably. If the global health community fails to support the WHO, the gap left by the USA could lead to fragmented responses and reduced access to essential health services for the world’s most vulnerable populations.

## CONCLUSION: A CALL FOR GLOBAL SOLIDARITY IN HEALTH GOVERNANCE

The USA’s decision to withdraw from the WHO is a pivotal moment in global health diplomacy, underscoring both the fragility of governance systems and the ongoing struggle to ensure health as a universal right. At this critical juncture, it is essential for the international community particularly countries with greater resources and influence to reaffirm their commitment to equity and multilateral collaboration. Renewed leadership that places health above political agendas, alongside genuine global solidarity, is needed to protect the global health agenda, reduce inequities, and build resilient systems capable of facing future crises.
